# Reverse Time‐to‐Death as Time‐Scale in Time‐to‐Event Analysis for Studies of Advanced Illness and Palliative Care

**DOI:** 10.1002/sim.10338

**Published:** 2025-01-23

**Authors:** Yin Bun Cheung, Xiangmei Ma, Isha Chaudhry, Nan Liu, Qingyuan Zhuang, Grace Meijuan Yang, Chetna Malhotra, Eric Andrew Finkelstein

**Affiliations:** ^1^ Programme in Health Services & Systems Research, Duke‐NUS Medical School National University of Singapore Singapore Singapore; ^2^ Centre for Quantitative Medicine, Duke‐NUS Medical School National University of Singapore Singapore Singapore; ^3^ Tampere Center for Child, Adolescent and Maternal Health Research Tampere University Tampere Finland; ^4^ Lien Center for Palliative Care, Duke‐NUS Medical School National University of Singapore Singapore Singapore; ^5^ Duke‐NUS AI + Medical Science Initiative, Duke‐NUS Medical School National University of Singapore Singapore Singapore; ^6^ Division of Supportive and Palliative Care National Cancer Centre Singapore Singapore Singapore; ^7^ Data and Computational Science Core National Cancer Centre Singapore Singapore Singapore

**Keywords:** advanced illness, palliative care, partial likelihood, time‐to‐event analysis, time‐varying confounding

## Abstract

Incidence of adverse outcome events rises as patients with advanced illness approach end‐of‐life. Exposures that tend to occur near end‐of‐life, for example, use of wheelchair, oxygen therapy and palliative care, may therefore be found associated with the incidence of the adverse outcomes. We propose a concept of reverse time‐to‐death (rTTD) and its use for the time‐scale in time‐to‐event analysis based on partial likelihood to mitigate the time‐varying confounding. We used data on community‐based palliative care uptake (exposure) and emergency department visits (outcome) among patients with advanced cancer in Singapore to illustrate. We compare the results against that of the common practice of using time‐on‐study (TOS) as time‐scale. Graphical analysis demonstrated that cancer patients receiving palliative care had higher rate of emergency department visits than non‐recipients mainly because they were closer to end‐of‐life, and that rTTD analysis made comparison between patients at the same time‐to‐death. In analysis of a decedent cohort, emergency department visits in relation to palliative care using TOS time‐scale showed significant increase in hazard ratio estimate when observed time‐varying covariates were omitted from statistical adjustment (% change‐in‐estimate = 16.2%; 95% CI 6.4% to 25.6%). There was no such change in otherwise the same analysis using rTTD (% change‐in‐estimate = 3.1%; 95% CI ‐1.0% to 8.5%), demonstrating the ability of rTTD time‐scale to mitigate confounding that intensifies in relation to time‐to‐death. A similar pattern was found in the full cohort. Simulations demonstrated that the proposed method had smaller relative bias and root mean square error than TOS‐based analysis. In conclusion, use of rTTD as time‐scale in time‐to‐event analysis provides a simple and robust approach to control time‐varying confounding in studies of advanced illness, even if the confounders are unmeasured.

## 1 Introduction

Advanced illness imposes substantial suffering on patients and healthcare costs on society, and the burdens are projected to increase rapidly in the next few decades [1]. Palliative care aims to improve quality of life of patients and their families who are facing challenges arising from advanced illness. It is hypothesized that palliative care can also reduce acute healthcare utilization that is not effective in promoting well‐being and therefore reduce healthcare costs [2, 3]. However, there has been limited evidence about such benefits [2, 4]. It was suggested that the differences in palliative care delivery in trial setting and real‐world setting led to under‐estimation of the effect of palliative care by randomized trials [5]. Observational studies, possibly using real‐world data, may play an important role in the evaluation.

Multivariable regression and propensity score methods have been used in a multitude of observational studies of emergency department (ED) visits, hospital admissions and hospital cost, including in the palliative care setting [6]. However, both methods rely on a critical assumption of no unobserved confounders, which is difficult to ascertain [7]. A recent systematic review maintained that while there “exists a large volume of studies using multivariable regression or propensity score approaches to control for observed confounding, … there has been insufficient attention paid to unobserved confounding and selection bias.” [6] An alternative approach that may handle unobserved confounding is difference‐in‐difference analysis, which compares rates of change in outcomes over time between populations that do and do not experience introduction of a policy intervention [8, 9].

In the setting of advanced illness, studies of exposures that tend to occur near end‐of‐life, such as use of wheelchair, oxygen therapy and palliative care, may suffer a high level of time‐varying confounding. For example, as a patient's health condition deteriorates near end‐of‐life, increase in symptom burden and decline in functional status may lead to higher level of utilization of both palliative and acute care [10]. If palliative care is analyzed as the exposure and ED visits as the outcome event in time‐to‐event analysis, the hazard ratio (HR) estimate would be biased upward due to the confounding [11, 12]. The deterioration in health condition also signifies the beginning of the final stretch of lifespan [11, 13]. This time‐varying confounding cannot be removed by adjustment or matching for time‐constant covariates. Moreover, data capture of time‐varying confounders such as palliative care needs is not commonly available in real‐world data and patients may be too ill to respond to survey assessment.

The Cox model and its extensions are major candidates in time‐to‐event analysis. The Cox model is for analysis of a single episode of an outcome event; the Andersen‐Gill (AG) model is one of its extensions suitable for analysis of multiple episodes of non‐terminal outcomes such as ED visits [14, 15]. We refer to them as Cox‐type models collectively. They allow researchers to choose the time‐scale according to context, though time‐on‐study (TOS, or *t* in statistical notation) may be employed without deliberation [16, 17]. The influence of the chosen time‐scale variable and its correlates on the outcome event rate is canceled out in the partial likelihood of the models. The potential time‐varying confounding related to the time‐scale is therefore non‐parametrically adjusted for. It is recommended that the time dimension that has the strongest relationship with the outcome should be chosen as the time‐scale [16]. There is strong evidence that age is a better choice for time‐scale when it has strong impact on the outcomes [17, 18]. In studies of infectious diseases, using calendar time as time‐scale has the advantage of controlling the confounding by seasonality or changing incidence [16, 19]. In studies of advanced illness, time‐to‐death (TTD) is a strong correlate of many outcomes such as symptom severity, functional decline and healthcare utilization [10, 11]. This motivated our research.

We propose “reverse time‐to‐death” (rTTD, or $t^{*}$ in statistical notation) as the time‐scale in Cox‐type models in studies of exposures that tend to occur near end‐of‐life. This serves as a proxy of observed or unobserved time‐varying confounders that intensify as patients approach end‐of‐life and mitigates their confounding effects. This analytic strategy is straight‐forward in studies of decedents, which is common in advanced illness and palliative care research. We also consider a procedure to estimate expected time‐to‐death among patients who were alive at the end of the study period and use it in the rTTD analysis. In our empirical case study, we hypothesized that: (a) Analysis of ED visits in relation to palliative care uptake using TOS as time‐scale would give larger HR than rTTD, representing a higher level of uncontrolled confounding in the former analysis. (b) Analysis with TOS as time‐scale would show larger changes in HR estimates when observed time‐varying covariates are omitted from statistical adjustment than analysis with rTTD, representing the ability of rTTD time‐scale to control unobserved confounding that intensifies in relation to time‐to‐death. We report a simulation study that compares the performance of rTTD and TOS in the present context.

## 2 Methods

### 2.1 Reverse Time‐to‐Death as Time‐Scale

**FIGURE 1:**
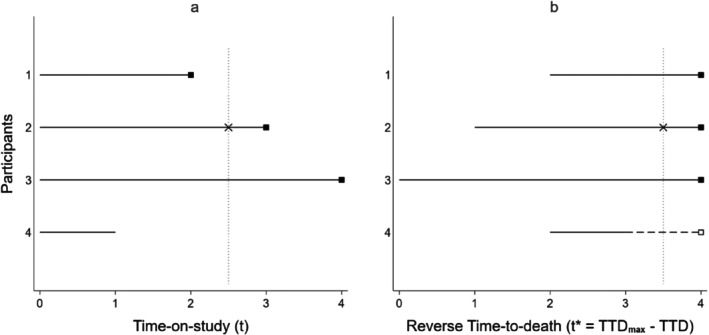
Illustration of (a) time‐on‐study (*t*) and (b) reverse time‐to‐death $\left(t^{*}\right)$ as time‐scales in Cox‐type models. ■ observed time of death; × observed time of outcome event; □ estimated time of death; solid line: Actual follow‐up time; dashed line: Time between end of actual follow‐up and estimated time of death; intersections of dotted line and solid lines indicate “at risk” persons at the event time.

Suppose the first three of *N* hypothetical study participants died at 2, 3 and 4 years after study enrolment, and the fourth participant was followed for 1 year and then dropped out (censored). Their follow‐up times are shown in Figure 1a using TOS as the time‐scale. For visual clarity, we plot only the first four of *N* participants in this figure.

All participants entered the study at time *t* = 0. Furthermore, participant 2 had an outcome event at *t* = 2.5 years. This event time contributes to the Cox‐type model's partial likelihood (*PL*) through [16]:

$$\begin{array}{cc} \mathrm{PL}\left(t=2.5\right) & =\frac{\lambda \left(t=2.5\right)\exp\left(\beta X_{2}\right)}{\lambda \left(t=2.5\right)\exp\left(\beta X_{2}\right)+\lambda \left(t=2.5\right)\exp\left(\beta X_{3}\right)+\ldots }\\ & =\frac{\exp\left(\beta X_{2}\right)}{\exp\left(\beta X_{2}\right)+\exp\left(\beta X_{3}\right)+\ldots } \end{array}$$

where λ(t) is the baseline hazard at time *t*, $X_{i}$ is a column vector of covariate values of participant *i*, β is a row vector of log(HR) to be estimated, and “…” represents the contributions to PL(t=2.5) by the other participants who were at risk at *t* = 2.5 (not shown in figure). In the above annotation, “baseline” means people whose observed covariate values are all zero. Cox‐type models estimate β by maximizing the logarithm of the model *PL*, which is the product of all *PL(t)* arising from the event times.

The intersections of the dotted line and solid lines in Figure 1a indicate “at risk” persons at the event time. Participants 1 and 4 were not included in the denominator of PL(t=2.5) because they were not at risk at this time. The baseline hazard at *t* = 2.5, λ(t=2.5), is canceled out in PL(t=2.5) as it appears in both the numerator and denominator [16, 18]. In words, when participant 2 had an event at 2.5 years after enrolment, his/her hazard is compared with that of participant 3 and other people (if any) who were also at risk at 2.5 years after enrolment. Therefore, the influence of TOS and its correlates is canceled out. However, at the time of the event participant 2 was only half a year from death, whereas participant 3 still had 1.5 years to go. They were not comparable in terms of TTD and time‐varying covariates that change sharply in relation to TTD.

Suppose we used the method to be discussed in the next section to estimate the survival time for participants whose survival time was censored, and the estimated survival time for participant 4 was 2 years. Figure 1b right‐aligns the follow‐up time. Time from censoring to estimated time of death was indicated by a dashed line. TTD is a time‐varying covariate that represents the duration from a point in time during a participant's follow‐up to the participant's time of death. The use of the rTTD time‐scale requires (observed or estimated) TTD at the time of study enrolment of all participant. We define:

$$t_{i}^{*}={\mathrm{TTD}}_{\max}-{\mathrm{TTD}}_{i}$$

where ${\mathrm{TTD}}_{i}$ is the *i*‐th participant's TTD at study enrolment, ${\mathrm{TTD}}_{\max}$ is the maximum of all ${\mathrm{TTD}}_{i}$, and $t_{i}^{*}$ is the *i*‐th participant's time at entry on the rTTD time‐scale. Suppose participant 3 had the longest time‐to‐death, so ${\mathrm{TTD}}_{\max}=4$. All participants who had observed survival time exit from the study at ${\mathrm{TTD}}_{\max}$. Participants who had censored survival time exit at $t_{i}^{*}$ plus the participant's follow‐up duration. Furthermore, we use ${\mathrm{TTD}}_{i}\left(t\right)$ to denote time‐to‐death values (in days) of the *i*‐th participant that are changing over time. Entry to the study is now staggered. Statistical software like Stata and R allow staggered entry. We used Stata's *stcox* program [20].

Participant 2 now has an outcome event at $t^{*}=3.5$. This event time contributes to the *PL* through:

$$\begin{array}{cc} & \mathrm{PL}\left(t^{*}=3.5\right)\\ & =\frac{\lambda \left(t^{*}=3.5\right)\exp\left(\beta X_{2}\right)}{\lambda \left(t^{*}=3.5\right)\exp\left(\beta X_{1}\right)+\lambda \left(t^{*}=3.5\right)\exp\left(\beta X_{2}\right)+\lambda \left(t^{*}=3.5\right)\exp\left(\beta X_{3}\right)+\ldots }\\ & =\frac{\exp\left(\beta X_{2}\right)}{\exp\left(\beta X_{1}\right)+\exp\left(\beta X_{2}\right)+\exp\left(\beta X_{3}\right)+\ldots } \end{array}$$

Participant 1 is now included in the denominator of $\mathrm{PL}\left(t^{*}=3.5\right)$ as s/he was at risk at $t^{*}=3.5$. Participant 4 is not included because s/he had left the study at $t^{*}=3$. However, if there were participants who had outcome events between $t^{*}=$ 2 and 3, participant 4 would be in the denominator of $\mathrm{PL}\left(t^{*}\right)$ at these event times. The hazard at this time‐to‐death, $\lambda \left(t^{*}=3.5\right)$, is canceled out in the *PL*. Therefore, the impact of time‐to‐death and its correlates on the outcome is eliminated.

In short, when participant 2 had an event half a year before death, his/her hazard of the outcome event is compared with that of people who were also at half a year before death. Using rTTD as time‐scale helps to compare like with like in terms of time‐to‐death and its associated time‐varying covariate values.

### 2.2 Estimation of Time‐to‐Death

Studies of decedents are common in research on advanced illness and palliative care, for example, the population‐based Ontario and Belgium decedent cohorts [21, 22]. The rTTD method is straight‐forward in this case.

The Buckley‐James (BJ) method is a distribution‐free method for estimation of expected survival time for censored observations given a dataset that includes both censored and observed survival times and observed covariates [23, 24]. It is implemented in statistical software such as Stata and R. We used Stata's *buckley* program [25]. The robustness may be low if a large proportion of observations is censored. Heller and Simonoff recommended the use of BJ method when censoring proportion is smaller than 40% [26]. Considering the paucity of model diagnostics for the method, Stare et al. recommended a more stringent criterion of < 20% [24]. The BJ method is suitable in studies of advanced illness where few patients survived despite its limitation in broader applications.

### 2.3 Case Study: Healthcare Utilization in Stage IV Cancer Patients

The Cost of Medical Care of Patients with Advanced Serious Illness in Singapore (COMPASS) is a prospective cohort study of 600 adult patients (age ≥ 21) with stage IV solid cancer, recruited between 2016 and 2018 from National Cancer Centre Singapore (NCCS) and National University Hospital System. Details of the study protocol have been published [27]. Findings on ED visits and other acute healthcare utilization in the last month of life has also been published [28]. Briefly, consented patients were interviewed every 3 months until death or 60 months post enrolment, whichever earlier. They also provided consent for access to their electronic health records (EHR) held by their healthcare providers, including acute care hospitals and community‐based palliative care providers. The EHR data covered till 31 December 2021. The study is approved by SingHealth Centralized Institutional Review Board (2015‐2781) and National University of Singapore Institutional Review Board (S‐20‐155).

For purpose of illustration of the use of rTTD time‐scale, we analyzed ED visits in relation to community‐based palliative care (PC for brevity), including home and day care and regardless of frequency/duration of utilization. In the present context, PC is a time‐varying exposure variable [16]. For example, if a patient started using PC in the mid‐point between study enrolment and death, the first half of the person‐time will be classified as unexposed and the second half as exposed.

Time‐constant covariates included age at enrolment, gender, type of cancer, MediFund status (an indicator of financial difficulty) and education. Time‐varying covariates from 3‐monthly interviews include the Physical Well‐being (PWB) and Functional Well‐being (FWB) scores of Functional Assessment of Cancer Therapy—General (FACT‐G), which are known predictors of cancer survival [29, 30]. Missing PWB and FWB values (16 and 17, respectively, out of totally 5499 survey questionnaires) were handled by last‐observation‐carried‐forward.

Since one person could have multiple ED visits, we used the AG model for the analysis and robust standard error for cluster data for inference [14, 15]. Bootstrapping was used to estimate confidence intervals for difference in HR estimates between different models, with persons as resampling units and 1000 replicates. For graphical presentation of hazard functions, we used kernel smoothing with boundary‐bias correction [20].

We began with analysis of patients who were deceased by end of 2021, which was the final time point the study team had permission to perform automated search for survival status in the databases. Then, for the full cohort analysis, we used the aforementioned time‐constant covariates and baseline PWB and FWB scores as predictors in the BJ method to estimate survival time for patients who were alive at the end of 2021. After 2021, manual review of medical records at NCCS found the date of death of 32 patients who died in 2022 or 2023. We used these 32 records to evaluate the accuracy of the BJ analysis, in which they were kept censored at the end of 2021, by comparing their observed and BJ‐estimated survival times.

### 2.4 Simulation Study

We conducted a series of simulation to evaluate and compare the methods. Each simulated dataset had a sample size of 600, same as the case study. The simulation parameters were also set to resemble the case study in terms of follow‐up time, treatment rate and event rate.

Time‐constant covariates, $x_{1},x_{2}$, and $x_{3}$, were generated from a multivariate normal distribution, each with mean = 0 and standard deviation = 1. In the main set of simulation scenarios, the correlations between them were $\rho_{1,2}=\rho_{1,3}=\rho_{2,3}=$ 0.5. Mortality rate was based on a Weibull distribution with hazard increasing over time:

$$M_{i}\left(t\right)=3t^{2}\exp\left(-18+x_{1,i}\right)$$

Each person's follow‐up time was partitioned into daily units and time‐to‐death in days, TTD(t), was created. A time‐varying confounder, z(t), was generated with mean = 0, standard deviation = 1, and correlation with TTD(t) being *r* = 0.9, 0.5 or 0.1, resembling a declining quality‐of‐life z‐score.

We set two versions of the hazard functions for treatment (exposure) initiation and outcome events. They had either a piecewise‐linear or linear relation with z(t). The hazard functions for treatment initiation was:

$$h_{i}^{\mathrm{trt}}\left(t\right)=\exp\left(-8+f\left[z_{i}\left(t\right)\right]+x_{2,i}\right)$$

It generated treatment initiation time ${\mathrm{trt}}_{i}$. Then, ${\mathrm{PC}}_{i}\left(t\right)=1\;\text{if}\;t>{\mathrm{trt}}_{i}$; ${\mathrm{PC}}_{i}\left(t\right)=0$ otherwise. The hazard function for outcome events was given by:

$$\lambda_{i}\left(t\right)=\exp\left(-8+\beta_{1}{\mathrm{PC}}_{i}\left(t\right)+f\left[z_{i}\left(t\right)\right]+x_{3,i}\right)$$

We considered treatment effect $\mathrm{HR}=\exp\left(\beta_{1}\right)=$ 0.5, 1.0, or 2.0. In the data generating processes that had piecewise linear and linear relation, respectively,

$$f\left[z_{i}\left(t\right)\right]=-0.5z_{i}\left(t\right)-I\left[z_{i}\left(t\right)\leq -1\;\right]2z_{i}\left(t\right)$$

where I[⋅] was an indicator function, and

$$f\left[z_{i}\left(t\right)\right]=-2z_{i}\left(t\right)$$

The hazards of treatment initiation and outcome events both increased with time due to their relation with z(t).

Time‐constant confounding arose due to correlation between $x_{2}$ and $x_{3}$. We fitted AG models using rTTD and TOS time‐scale with adjustment for (1) no covariates, (2) only time‐constant covariate $x_{3}$, and (3) $x_{3}$ and z(t). Mode (1) represented a worst‐case scenario of no measured covariates. Model (3) represented a best‐case scenario that z(t) was continuously measured without error. For TOS time‐scale, we also fitted a model (4) with $x_{3}$ and TTD(t) as covariates. Comparison of model (2) under rTTD and model (4) under TOS time‐scale shed light to the performance of using rTTD to non‐parametrically absorb time‐varying confounding into Cox‐type models' unspecified baseline versus directly including TTD(t) as a time‐varying covariate in TOS analysis. All covariates were entered on one degree of freedom.

In further simulation to explore whether correlation with mortality had an impact on the performance of the methods, we set $\rho_{1,2}=\rho_{1,3}=$ 0 or 0.25 in addition. To explore whether the time‐scales performed differently in the presence of only time‐constant confounding, we changed the hazard function for outcome events to:

$$\lambda_{i}\left(t\right)=\exp\left(-6+\beta_{1}{\mathrm{PC}}_{i}\left(t\right)+x_{3,i}\right)$$

We used 1000 replicates for each simulation scenario, and report the relative bias (Rel. Bias, %), coverage probability (CP, %) of 95% confidence interval, and root mean square error (RMSE).

## 3 Results

### 3.1 Case Study

Two of 600 patients were excluded from analysis due to missing covariate data. By the end of 2021, 429 of 598 patients had died. Among the decedents, 207 patients had ever used PC during the study period. There were 885 ED visits and 651.5 person‐years of observation (Table 1). The incidence rate (number of ED visits per person‐year) was lower among patients who did not use PC than patients who did (1.19 versus 1.55). Among the latter group, incidence rate was lower before their starting PC than after (1.06 versus 3.10).

**TABLE 1:** Incidence rate (number of events/person‐years) of emergency department visit, by exposure to palliative care.

			PC users		
Sample	Overall	Non‐PC users	All time	Before PC	After PC
Decedents (*n* = 429)	1.36 (885/651.5)	1.19 (407/343.4)	1.55 (478/308.1)	1.06 (247/233.5)	3.10 (231/74.6)
Full cohort (*n* = 598)	0.79 (1147/1452.9)	0.58 (664/1140.4)	1.55 (483/312.6)	1.05 (248/236.3)	3.08 (235/76.3)

Abbreviation: PC: palliative care.

**FIGURE 2:**
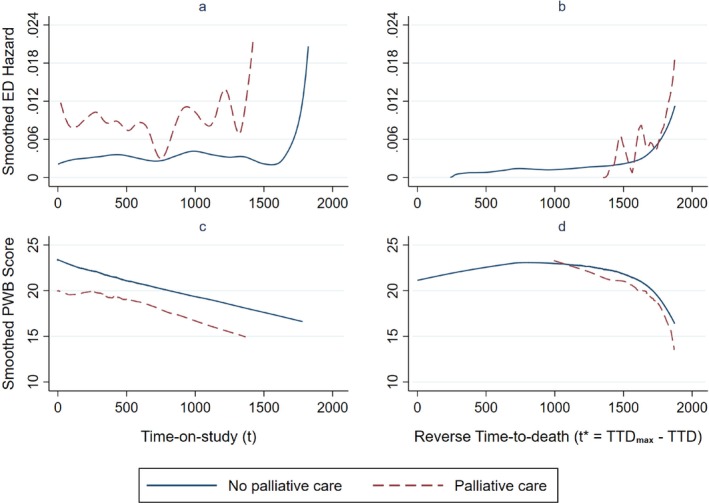
Smoothed estimates of hazard of emergency department (ED) visit and smoothed mean Physical Well‐being (PWB) score by time‐scale and exposure to palliative care; decedents only. [Colour figure can be viewed at wileyonlinelibrary.com]

Figure 2a–d show the smoothed hazard estimates of ED visits and smoothed mean PWB scores among the decedents. Using TOS time‐scale, there was a wide gap in hazard of ED visits between person‐time exposed and unexposed to PC and the hazard was roughly stable over time except at the tail ends (Figure 2a). In contrast, using rTTD time‐scale reveals that the hazard increased as patients approached end‐of‐life and that person‐time on PC had higher hazard mainly because this was nearer end‐of‐life (Figure 2b). Comparing the estimates at the same time‐to‐death, the difference in hazard between person‐time on and not on PC was much smaller than in Figure 2a.

From the TOS perspective (Figure 2c), there was a gap of about three to four points in mean PWB score between PC and no PC. Using the rTTD perspective reveals that PWB scores were lower in person‐time on PC mainly because they were nearer end‐of‐life (Figure 2d). Given the same time‐to‐death, the difference in PWB between the two curves was only about one point. Statistical adjustment for PWB would therefore make a substantial impact on the HR estimates in analysis of ED utilization based on TOS but not rTTD. A similar pattern was seen in FWB scores (details not shown).

Among the decedents, without controlling for any covariates, the HRs were 2.71 and 1.31 with TOS and rTTD as time‐scale, respectively (Table 2). The difference in HR was 1.40 (95% CI 0.97 to 1.82). The finding was consistent with our first hypothesis that using TOS as time‐scale would give a higher HR estimate than rTTD. Adjustment for time‐constant covariates made little difference to the HR estimates. This trivial change‐in‐estimate should not be taken as evidence of no time‐constant confounding because unobserved time‐constant covariates may be dominant.

**TABLE 2:** Hazard ratios of emergency department visit in person‐time exposed to palliative care versus unexposed, from Andersen‐Gill models using different time‐scales.

		Covariate adjustment					
		None		TCC only		TCC + TVC	
Sample	Time‐scale	HR	95% CI	HR	95% CI	HR	95% CI
Decedents	TOS	2.71	(2.14, 3.43)	2.72	(2.15, 3.44)	2.34	(1.85, 2.97)
(*n* = 429)	rTTD	1.31	(1.04, 1.66)	1.32	(1.05, 1.66)	1.28	(1.02, 1.60)
Full cohort	TOS	4.27	(3.38, 5.38)	4.20	(3.32, 5.32)	3.09	(2.45, 3.90)
(*n* = 598)	rTTD	2.12	(1.67, 2.69)	2.05	(1.61, 2.60)	1.85	(1.46, 2.34)

Abbreviations: HR: Hazard ratio; rTTD: Reverse time‐to‐death; TCC + TVC: Time‐constant and time‐varying covariates; see Section 2 for covariates included; TCC: Time‐constant covariates; TOS: Time‐on‐study.

The results were also consistent with our second hypothesis that omission of adjustment for time‐varying covariates would make larger change‐in‐estimate in analysis using TOS than rTTD. The adjustment led to difference in HR between 2.72 and 2.34 using TOS (% change‐in‐estimate = 16.2%; 95% CI 6.4% to 25.6%). In contrast, the adjustment only made a trivial difference in HR between 1.32 and 1.28 using rTTD (% change‐in‐estimate = 3.1%; 95% CI −1.6% to 8.6%).

In analysis of the full cohort, time‐to‐death was estimated using the BJ method for 169 patients who were alive at the end of 2021. They were mostly non‐PC users who had low ED utilization, leading to lower incidence of ED visits in the non‐PC users (0.58 per person‐year, Table 1) and higher HRs in the full cohort than decedents (Table 2). However, the pattern of HR estimates between the two time‐scales was similar to that in the decedent analysis.

The mean observed and estimated time‐to‐death of the set of 32 observations earmarked for evaluation of the BJ method were 5.53 and 6.29 years, respectively. The mean absolute difference was 1.0 year. Thus, the right‐alignment of the survivors' follow‐up times as illustrated in Figure 1b might have been somewhat inaccurate. This could generate residual confounding. This may explain why adjustment for time‐varying confounders led to a larger change in HR (2.05 vs. 1.85, % change‐in‐estimate = 10.8%; 95% CI 2.7% to 18.9%) in the rTTD analysis in the full cohort than in decedents only. Nevertheless, it was still much less than the change‐in‐estimate between 4.20 and 3.09 using TOS (% change‐in‐estimate = 35.9%; 95% CI 19.1% to 49.2%).

### 3.2 Simulation Study

Table 3 shows the results in scenarios of true HR = 0.5 and the time‐varying confounder, z(t), had piecewise linear relation with event hazard. Given the same covariates adjusted, using rTTD gave smaller relative bias, smaller RMSE and CP closer to target level than TOS. Even in the best‐case scenario (model 3) that z(t) was measured continuously without error, rTTD still performed better except when *r* = 0.1. Furthermore, even if TOS models included TTD(t) as a time‐varying covariate in addition to time‐constant covariate $x_{3}$ (model 4), it still had poorer performance than the rTTD model that adjusted only for $x_{3}$ (model 2). The relative merit of rTTD is most obvious when *r* = 0.9, but it remains visible even when *r* = 0.1. While rTTD tended to give much better estimation results than TOS, it still showed some degree of bias and CP could be much lower than target level, especially when *r* was small.

**TABLE 3:** Simulation results in scenarios of piecewise linear relation between time‐varying confounder [z(t)] and outcome event hazard; HR = 0.5; ρ = 0.5; $x_{3}$ is time‐constant covariate; correlation (*r*) between z(t) and time‐to‐death in days [TTD(t)] equals 0.9, 0.5 or 0.1.

			rTTD			TOS		
*r*	Model	Covariates	Rel. bias	CP	RMSE	Rel. bias	CP	RMSE
0.9	1	None	66.7%	4.1%	0.355	318.7%	0.0%	1.625
	2	$x_{3}$	8.7%	86.8%	0.076	131.2%	0.0%	0.765
	3	$x_{3}$ + z(t)	3.3%	92.4%	0.061	6.3%	89.5%	0.067
	4	$x_{3}$ + TTD(t)	NA	NA	NA	16.3%	70.6%	0.105
0.5	1	None	88.0%	0.0%	0.448	269.2%	0.0%	1.357
	2	$x_{3}$	37.4%	0.1%	0.193	120.4%	0.0%	0.610
	3	$x_{3}$ + z(t)	5.4%	88.6%	0.046	5.5%	87.5%	0.047
	4	$x_{3}$ + TTD(t)	NA	NA	NA	38.2%	1.1%	0.198
0.1	1	None	76.1%	0.0%	0.388	144.2%	0.0%	0.731
	2	$x_{3}$	48.6%	0.0%	0.248	60.8%	0.0%	0.310
	3	$x_{3}$ + z(t)	5.3%	86.1%	0.045	4.7%	88.1%	0.045
	4	$x_{3}$ + TTD(t)	NA	NA	NA	53.0%	0.1%	0.271

Abbreviations: CP: coverage probability of 95% confidence interval; Rel. bias: relative bias; RMSE: root mean square error.

Table 4 shows the results in scenarios that were otherwise the same as Table 3 but z(t) had a linear relation with event hazard. Having adjusted for no covariate (model 1) or only time‐constant covariate (model 2), models using rTTD out‐performed models using TOS. However, for this simple functional form of confounder effect, TOS model (4) with adjustment for TTD(t) and $x_{3}$ had relative bias, RMSE and CP similar to the rTTD model (2) that adjusted only for $x_{3}$.

**TABLE 4:** Simulation results in scenarios of linear relation between time‐varying confounder [z(t)] and outcome event hazard; HR = 0.5; ρ = 0.5; $x_{3}$ is time‐constant covariate; correlation (*r*) between z(t) and time‐to‐death in days [TTD(t)] equals 0.9, 0.5 or 0.1.

			rTTD			TOS		
*r*	Model	Covariates	Rel. bias	CP	RMSE	Rel. bias	CP	RMSE
0.9	1	None	58.4%	22.1%	0.320	247.3%	0.0%	1.271
	2	$x_{3}$	3.0%	94.7%	0.073	83.1%	4.4%	0.439
	3	$x_{3}$ + z(t)	1.5%	95.4%	0.070	1.5%	94.4%	0.070
	4	$x_{3}$ + TTD(t)	NA	NA	NA	2.8%	94.5%	0.073
0.5	1	None	63.3%	2.6%	0.332	176.4%	0.0%	0.899
	2	$x_{3}$	15.3%	73.1%	0.095	57.3%	2.5%	0.299
	3	$x_{3}$ + z(t)	3.2%	94.5%	0.053	2.8%	95.5%	0.052
	4	$x_{3}$ + TTD(t)	NA	NA	NA	15.1%	73.6%	0.094
0.1	1	None	50.9%	8.4%	0.270	96.3%	0.0%	0.498
	2	$x_{3}$	19.4%	57.4%	0.113	24.2%	46.6%	0.137
	3	$x_{3}$ + z(t)	2.0%	94.0%	0.051	1.9%	93.8%	0.052
	4	$x_{3}$ + TTD(t)	NA	NA	NA	20.1%	57.0%	0.118

Abbreviations: CP: coverage probability of 95% confidence interval; Rel. bias: relative bias; RMSE: root mean square error.

Similar results were obtained for HR = 1 and 2, and $\rho_{1,2}=\rho_{1,3}=$ 0.25 and 0 (Data S1: Tables S1–S5).

We also explored the adjustment or omission of a time‐constant confounder in the absence of time‐varying confounding. When the time‐constant confounder was adjusted for, models using the two time‐scales gave similar results (Table 5). However, analysis using rTTD was more robust to omission of time‐constant confounder than TOS. This difference diminished as the correlation between mortality, treatment and outcome events diminished. It is an unexpected finding that choice of time‐scales may affect model robustness in relation to omission of time‐constant covariates. The reasons of this is unclear. In Data S2, we provide some tentative explanation.

**TABLE 5:** Simulation results for scenarios with time‐constant covariate ($x_{3}$) only; HR = 0.5; *r* = 0.5; correlation (ρ) between time‐constant covariates of mortality and outcome event hazard equals 0.5, 0.25, or 0.

			rTTD			TOS		
ρ	Model	Covariates	Rel. bias	CP	RMSE	Rel. bias	CP	RMSE
0.5	1	None	42.7%	27.2%	0.234	73.4%	2.2%	0.385
	2	$x_{3}$	0.9%	95.1%	0.053	0.7%	95.6%	0.051
0.25	1	None	53.9%	13.4%	0.290	69.0%	3.9%	0.364
	2	$x_{3}$	0.7%	94.8%	0.052	0.9%	94.9%	0.050
0	1	None	62.4%	7.1%	0.332	58.2%	9.2%	0.310
	2	$x_{3}$	1.2%	94.5%	0.050	0.9%	94.5%	0.049

Abbreviations: CP: coverage probability of 95% confidence interval; Rel. bias: relative bias; RMSE: root mean square error.

## 4 Discussion

The choice of time‐scale in Cox‐type models offers a simple and robust way to control time‐varying confounding. A general recommendation is to choose the time dimension that has the strongest relationship with the outcome [16, 18]. In advanced illness, the level of many outcomes change sharply near end‐of‐life, making time‐to‐death a suitable choice. In clinical practice, clinicians do not know TTD in advance so they cannot personalize healthcare accordingly. But in research on advanced diseases we know TTD at the time of data analysis. The present article is relevant only to research practice.

Some advanced illness studies involve decedents only, but some also involve patients who were alive at the end‐of‐study. The Buckley‐James method can be used to estimate their survival times. The robustness of the analysis is affected by the proportion of participants with censored survival time. Studies of advanced illness with only a small proportion of censored observations, preferably < 20% and at most 40% [24, 26], may consider using this approach.

The proposed method should be employed only if date of death is captured reliably. It is naturally applicable to decedent cohorts. It is also applicable to cohort studies in which most cohort members have observed survival time, such that the use of the BJ method for estimation of expected survival time is robust. Furthermore, the observed at‐risk time of the cohort members who have censored survival time should to some extent overlap with the observed at‐risk time of the decedents, as exemplified in Figure 1b. Otherwise, the survivors would not be included in the risk‐sets at event times near ${\mathrm{TTD}}_{\max}$ that (palliative care) exposure is likely and would risk becoming non‐informative in the models. The benefits of using the rTTD time‐scale is clearest when time‐varying confounders are strongly correlated with TTD(t). However, simulation in which the correlation was only 0.1 showed no harm in using rTTD as compared to TOS. So it is appropriate to use rTTD provided that there is clinical/epidemiology expectation that (unobserved) time‐varying confounders are correlated with time‐to‐death despite uncertainty of the magnitude.

When TTD is known, for example, in decedent cohorts, it is possible to use TOS time‐scale and include TTD(t) as an observed time‐varying covariate in the analysis. However, this would require specification of some functional form for this covariate‐outcome relationship, for example, assuming a linear relationship or using step‐functions. In contrasts, the use of the proposed rTTD time‐scale non‐parametrically adjusts for TTD(t) by canceling its effect out in the numerator and denominator of the partial likelihood, as demonstrated in Section 2.1. It is a robust and parsimonious approach. This is borne out in comparing the simulation results on rTTD model (2) and TOS model (4) in scenarios where the time‐varying confounder had a piecewise linear versus a linear form of effect on event hazard. The same consideration similarly holds for, for example, using calendar time as time‐scale in studies of diseases with strong seasonality even though it is possible to include season as an observed time‐varying covariate [16, 19].

There are multiple reasons why in the case study adjustment for observed time‐varying confounders made the results between TOS and rTTD relatively similar but a substantial difference remains. First, there were unobserved time‐varying confounders. For example, we did not have measures of physical health deterioration. Second, self‐reported measures like PWB and FWB were subject to sizable measurement error [31]. Third, for practical reasons, time‐varying covariates are usually measured at regular intervals instead of continuously. In the case study, their time‐varying values were the values at the latest 3‐monthly survey date. Fourth, the functional forms of the relationship between these covariates and the outcome could have been mis‐specified.

As seen in the simulation, the use of rTTD tends to give improved estimation results as compared to TOS in the context of advanced disease and palliative care, but it does not guarantee completely correct point or interval estimation, which is dependent on factors including magnitude of correlation between time‐varying confounder. While the rTTD can mitigate confounding, it does not entirely remove the need for collection of data on time‐varying confounders if that is possible. Taken together, adjustment for observed (time‐varying) covariates and the use of rTTD are complementary to each other.

This study has focused on time‐varying confounding. It is important to also search for better approaches to handle time‐constant confounding. We do not interpret the presented analytic results from COMPASS as an indication of palliative care leading to higher rate of ED visits because unobserved time‐constant confounders such as psychosocial factors is still an issue [32, 33, 34]. The prior event rate ratio approach that is gaining popularity in biopharmaceutical research is basically a ratio‐of‐ratio analysis [35, 36]. Conceptually this is similar to difference‐in‐difference analysis that is popular in policy research [8, 9]. This method aims to control the impact of observed or unobserved time‐constant confounders in time‐to‐event analysis. Combined use of reverse time‐to‐death and prior event rate ratio appears promising and is an area for future research. Furthermore, the relation between the choice of time‐scale and robustness to omission of time‐constant covariate is currently unclear. It can also benefit from further research.

## Disclosure

Any opinions, findings and conclusions or recommendations expressed in this material are those of the authors and do not reflect the views of Ministry of Health/National Medical Research Council, Singapore, Singapore Millennium Foundation, or Lien Centre for Palliative Care.

## Conflicts of Interest

The authors declare no conflicts of interest.

## Supporting information


**Data S1** Supporting Information.

**Data S2** Supporting Information.

## Data Availability

Stata codes illustrating the use of rTTD and TOS time‐scales for the COMPASS study and Stata codes for the simulation are available from https://github.com/cheungyb/rTTD. COMPASS study data are not publicly available due to restrictions on distribution of electronic health record data. Data are however available from the authors upon reasonable request and permission of SingHealth and National University Health Systems and institutional review board approval from the requesting institution.
