# Supplementary material for: Reverse Time‐to‐Death as Time‐Scale in Time‐to‐Event Analysis for Studies of Advanced Illness and Palliative Care
Source: Stat Med. 2025 Jan 23;44(3-4):e10338. doi: 10.1002/sim.10338 (PMC11755714; doi:10.1002/sim.10338)
Supplement: Supplementary file 2 — Data S2 Supporting Information. [file SIM-44-0-s002.pdf]

Online Supplementary Material 1

Additional simulation results: Tables S1 to S5 (pages 2-6).

Rel. Bias: relative bias
CP: coverage probability of 95% confidence interval
RMSE: root mean square error.

x_3: time-constant confounder
$z(t)$: time-varying confounder
TTD(t): time-to-death in days

Online Supplementary Material 2

Additional comments on and illustration of impact of time-scales on robustness to omission of time-constant covariate: Figure S1 (page 7).

Table S1. Simulation results in scenarios of piecewise linear relation between $z(t)$ and outcome event hazard; HR = 1; ρ = 0.5; correlation (r) between $z(t)$ and TTD(t) = 0.9, 0.5 or 0.1.

r	Model	Covariates	rTTD			TOS		
			Rel. Bias	CP	RMSE	Rel. Bias	CP	RMSE
0.9	1	None	58.4%	2.3%	0.629	301.6%	0.0%	3.102
	2	x_3	4.6%	89.4%	0.113	124.2%	0.0%	1.288
	3	x_3 + $z(t)$	-0.4%	92.8%	0.096	1.8%	92.1%	0.097
	4	x_3 + TTD(t)	NA	NA	NA	10.9%	74.8%	0.157
0.5	1	None	64.6%	0.0%	0.667	224.4%	0.0%	2.275
	2	x_3	22.8%	8.6%	0.242	98.1%	0.0%	1.000
	3	x_3 + $z(t)$	-2.8%	92.3%	0.064	-2.2%	92.7%	0.062
	4	x_3 + TTD(t)	NA	NA	NA	24.0%	8.5%	0.256
0.1	1	None	51.4%	0.3%	0.534	111.1%	0.0%	1.136
	2	x_3	29.2%	1.0%	0.304	41.7%	0.2%	0.432
	3	x_3 + $z(t)$	-3.8%	88.5%	0.067	-4.7%	84.4%	0.075
	4	x_3 + TTD(t)	NA	NA	NA	34.7%	1.1%	0.362

Table S2. Simulation results in scenarios of linear relation between $z(t)$ and outcome event hazard; HR = 1; ρ = 0.5; correlation (r) between $z(t)$ and TTD(t) = 0.9, 0.5 or 0.1.

r	Model	Covariates	rTTD			TOS		
			Rel. Bias	CP	RMSE	Rel. Bias	CP	RMSE
0.9	1	None	54.0%	13.5%	0.600	239.5%	0.0%	2.485
	2	x_3	1.3%	94.6%	0.115	81.5%	0.7%	0.861
	3	x_3 + $z(t)$	-0.3%	94.7%	0.111	-0.2%	94.7%	0.110
	4	x_3 + TTD(t)	NA	NA	NA	1.0%	94.4%	0.114
0.5	1	None	52.1%	3.5%	0.557	159.3%	0.0%	1.639
	2	x_3	8.9%	81.1%	0.132	50.2%	1.2%	0.528
	3	x_3 + $z(t)$	-1.5%	94.2%	0.084	-1.2%	93.5%	0.086
	4	x_3 + TTD(t)	NA	NA	NA	9.0%	82.2%	0.134
0.1	1	None	41.0%	10.2%	0.444	84.6%	0.1%	0.881
	2	x_3	13.0%	65.6%	0.162	18.5%	48.9%	0.214
	3	x_3 + $z(t)$	-1.6%	93.9%	0.079	-1.7%	93.8%	0.082
	4	x_3 + TTD(t)	NA	NA	NA	14.4%	63.3%	0.177

Table S3. Simulation results in scenarios of piecewise linear relation between $z(t)$ and outcome event hazard; HR = 2; ρ = 0.5; correlation (r) between $z(t)$ and TTD(t) = 0.9, 0.5 or 0.1.

r	Model	Covariates	rTTD			TOS		
			Rel. Bias	CP	RMSE	Rel. Bias	CP	RMSE
0.9	1	None	49.4%	3.6%	1.043	283.6%	0.0%	5.752
	2	x_3	0.3%	94.4%	0.161	117.7%	0.0%	2.400
	3	$x_3 + z(t)$	-4.3%	88.3%	0.175	-2.7%	89.4%	0.158
	4	x_3 + TTD(t)	NA	NA	NA	4.9%	91.8%	0.197
0.5	1	None	41.0%	1.8%	0.851	177.9%	0.0%	3.587
	2	x_3	7.4%	78.2%	0.191	74.5%	0.0%	1.511
	3	$x_3 + z(t)$	-12.4%	31.7%	0.266	-11.0%	42.5%	0.241
	4	x_3 + TTD(t)	NA	NA	NA	9.2%	6.7%	0.226
0.1	1	None	27.3%	12.9%	0.582	77.8%	0.0%	1.585
	2	x_3	9.7%	61.3%	0.229	21.8%	11.4%	0.462
	3	$x_3 + z(t)$	-14.5%	14.5%	0.305	-15.4%	14.2%	0.323
	4	x_3 + TTD(t)	NA	NA	NA	15.8%	32.7%	0.348

Table S4. Simulation results in scenarios of linear relation between $z(t)$ and outcome event hazard; HR = 2; ρ = 0.5; correlation (r) between $z(t)$ and TTD(t) = 0.9, 0.5 or 0.1.

r	Model	Covariates	rTTD			TOS		
			Rel. Bias	CP	RMSE	Rel. Bias	CP	RMSE
0.9	1	None	51.3%	11.3%	1.098	236.8%	0.0%	4.823
	2	x_3	-0.2%	93.8%	0.185	82.2%	0.0%	1.692
	3	x_3 + $z(t)$	-1.5%	93.7%	0.185	-1.1%	93.3%	0.183
	4	x_3 + TTD(t)	NA	NA	NA	-0.3%	94.0%	0.187
0.5	1	None	42.3%	7.7%	0.893	144.5%	0.0%	2.934
	2	x_3	2.8%	92.1%	0.164	43.2%	1.0%	0.895
	3	x_3 + $z(t)$	-5.9%	85.5%	0.181	-4.9%	87.6%	0.169
	4	x_3 + TTD(t)	NA	NA	NA	3.3%	92.0%	0.171
0.1	1	None	30.7%	23.8%	0.667	71.6%	0.1%	1.476
	2	x_3	5.3%	88.6%	0.181	11.0%	71.2%	0.276
	3	x_3 + $z(t)$	-6.6%	80.9%	0.186	-7.0%	80.1%	0.194
	4	x_3 + TTD(t)	NA	NA	NA	7.2%	85.5%	0.214

Table S5. Simulation results in scenarios of piecewise linear relation between $z(t)$ and outcome event; HR = 0.5; r = 0.5; correlation (ρ) between time-constant covariates of mortality and outcome event = 0.5, 0.25 or 0.

ρ	Model	Covariates	rTTD			TOS		
			Rel. Bias	CP	RMSE	Rel. Bias	CP	RMSE
0.5	1	None	88.0%	0.0%	0.448	269.2%	0.0%	1.357
	2	x_3	37.4%	0.7%	0.193	120.4%	0.0%	0.610
	3	$x_3 + z(t)$	5.4%	88.6%	0.046	5.5%	87.5%	0.047
	4	x_3 + TTD(t)	NA	NA	NA	38.2%	1.1%	0.198
0.25	1	None	96.4%	0.0%	0.490	251.1%	0.0%	1.267
	2	x_3	36.3%	0.8%	0.187	128.5%	0.0%	0.650
	3	$x_3 + z(t)$	5.7%	87.1%	0.047	5.6%	86.7%	0.046
	4	x_3 + TTD(t)	NA	NA	NA	37.5%	0.8%	0.194
0	1	None	103.1%	0.0%	0.524	225.9%	0.0%	1.140
	2	x_3	34.9%	1.2%	0.181	133.5%	0.0%	0.675
	3	$x_3 + z(t)$	5.7%	86.2%	0.047	5.4%	87.2%	0.046
	4	x_3 + TTD(t)	NA	NA	NA	36.5%	0.7%	0.189

Online Supplementary Material 2

Additional comments on and illustration of impact of time-scales on robustness to omission of time-constant covariate.

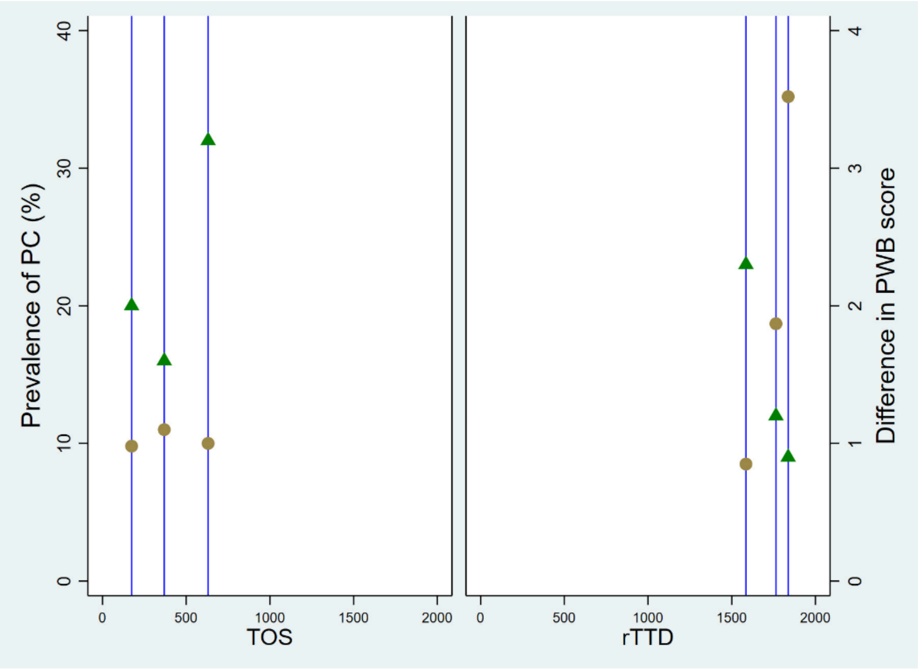

Figure S1. Prevalence of exposure (palliative care, PC; y-axis on the left) and difference in mean baseline Physical Well-being scores (PWB; y-axis on the right); TOS on left- and rTTD on right-hand-side panel. Brown dots (●): prevalence of exposure; green triangles (▲): difference in mean baseline PWB scores (non-PC minus PC); vertical blue lines: first, second and third quartiles of event times according to TOS or rTTD.

An intuitive explanation of the observation of rTTD being less affected by omission of adjustment for time-constant covariates is that, on the rTTD time-scale, prevalence of exposure (palliative care) and outcome event rate are high near end-of-life ($TTD_{max} = 1875$). As the exposure becomes more common, the exposed group becomes less selective and the difference in time-constant covariate values between the exposed and unexposed groups becomes smaller. This is illustrated in the right-hand-side panel of Figure S1 using the case study dataset, showing the higher prevalence of PC (9% to 35%) and smaller difference (non-PC minus PC) in mean of baseline PWB scores (0.9 to 2.3) at the first, second and third quartiles of event times on rTTD time-scale. In contrast, the left-hand-side panel of Figure S1 shows that on the TOS time-scale the prevalence of exposure was lower (10% to 11%, as patients initiated PC and exited from study at variable TOS time-points) and the difference in mean baseline PWB scores tended to be higher (1.6 to 3.2). As such, (omission of) adjustment for time-constant covariate may have bigger impact on TOS- than rTTD-based analysis.
